# Data Sharing: Convert Challenges into Opportunities

**DOI:** 10.3389/fpubh.2017.00327

**Published:** 2017-12-04

**Authors:** Ana Sofia Figueiredo

**Affiliations:** ^1^Department of Anesthesiology and Surgical Intensive Care Medicine, Medical Faculty Mannheim, University of Heidelberg, Mannheim, Germany; ^2^Institute for Experimental Internal Medicine, Medical Faculty, Otto-von-Guericke University, Magdeburg, Germany

**Keywords:** data sharing, data privacy, digital health, big data, FAIR guiding principles, open data

## Abstract

Initiatives for sharing research data are opportunities to increase the pace of knowledge discovery and scientific progress. The reuse of research data has the potential to avoid the duplication of data sets and to bring new views from multiple analysis of the same data set. For example, the study of genomic variations associated with cancer profits from the universal collection of such data and helps in selecting the most appropriate therapy for a specific patient. However, data sharing poses challenges to the scientific community. These challenges are of ethical, cultural, legal, financial, or technical nature. This article reviews the impact that data sharing has in science and society and presents guidelines to improve the efficient sharing of research data.

## Introduction

Scientific discovery needs support from research data. Data sharing provides others with access to that data. It avoids the generation of equivalent data sets, brings new perspectives from the re-analysis of the same data set and, in health care, can support diagnosis and treatment decisions. Nevertheless, data producers might be reluctant to share it in the first place. This is because data sharing poses challenges at diverse levels. These challenges are multifaceted and can be cultural, ethical, financial, and/or technical.

Although data sharing comes with specific costs, Roche and colleagues suggest changes that push up the relation between gain and cost of making scientific data available, for example, increase embargo flexibility or recognize the value of shared data (1). Embargo flexibility refers to the idea that shared metadata (data that provide information about the research data) may not be immediately available to others (embargo) and any delay in making these data available can vary (flexibility). Further on, the Research Data Alliance, an international organization created in 2013, develops the social and technical infrastructures that enable open sharing of data (2). Moreover, sharing metadata can improve the independent replication of research data and results.

The concept of open data shares the philosophy of open source (3) and open access (4). This way, open data can be freely re-used and re-distributed subject to a specific license. For example, the creative commons provide standardized licenses and tools to help establishing the conditions to reuse any type of creative work (5). In the particular case of research data, scientists have access not only to publications but also to the data involved in those studies.

**Figure 1:**
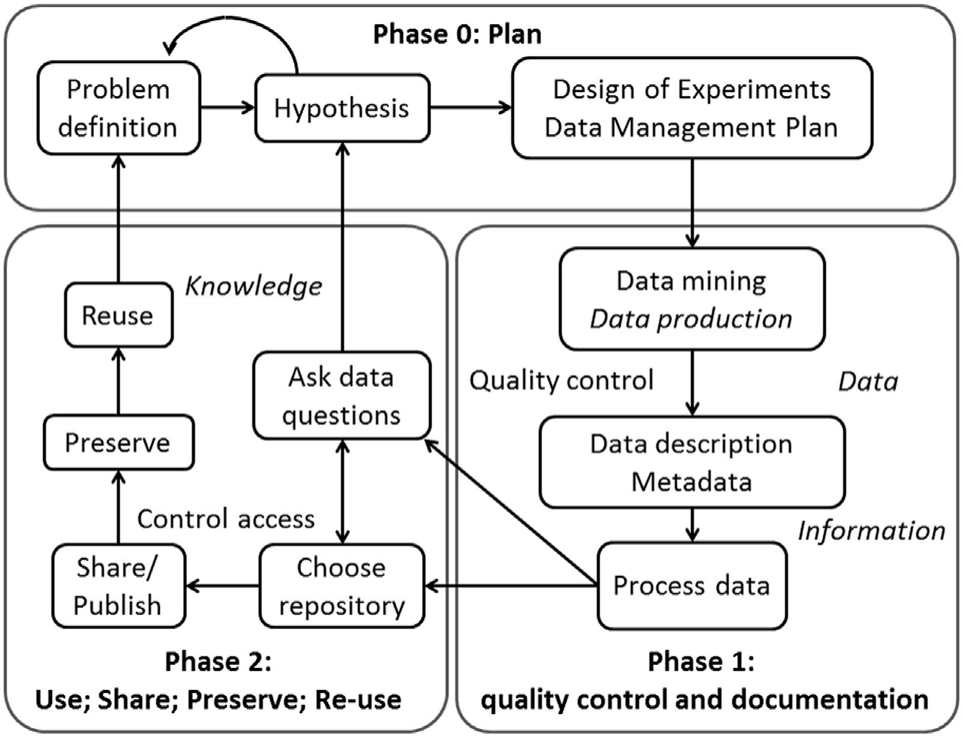
The life cycle of scientific data. Phase 0 refers to the planning: from the problem definition, scientists will generate a hypothesis and will develop the proper design of experiments and a data management plan. Phase 1: scientists will search in data repositories and in literature for answers to their questions and, if this is not enough, will produce the specific data set. Then, they will use specific metadata to structure/describe the data. Thereafter, this structured data can be processed. Phase 2 refers to the (re-)use, share, preservation, and data reusability. After processing, the scientists will ask specific questions concerning the data and will analyze it accordingly. They can also choose a repository to store the data. Then, they can share and/or publish the data in order to preserve it and allow others to reuse it (or reuse it themselves in the future). This allows for multiple analyses of the same data set.

Figure 1 represents the life cycle of research data, from the initial phase of planning to the phase of sharing and preserving.

In this review, we explore the challenges and opportunities behind data sharing and we go through specific steps that can turn research data amenable to share.

## Challenges

Although data sharing can benefit science and society, there are challenges behind the process of sharing data. These challenges are at the ethical/legal, cultural, financial, and/or technical levels.

Dealing with clinical trials and patients’ data raises ethical and legal issues related to data de-identification and their possible re-identification. In fact, OMICs data sets do not allow for a complete patient/donor anonymization and, therefore, revolutionize the traditional ethical and legal approaches on the usage of clinical data (6, 7). For example, the Personal Genome Project joins several sources of human data, donated by volunteers to improve scientific progress (6). Genome donation and open consent, together with controlled access to data and robust data warehousing facilities with the technical means to safeguard data and metadata can help overcome these issues (7). Therefore, technical solutions for data sharing encompass facilities for data storage, management, and analysis that are robust and reliable. Implementing these solutions and acquiring the expertise to do it comes with financial costs. Some laboratories might be reluctant to invest on data sharing, because these financial costs are high and might not have an immediate return. Also at the cultural level, the fear that competitors can come upon new findings first and that their data can be misused or misinterpreted may hamper data sharing. In this setting, education and engagement of the scientific community is essential.

## Opportunities

Life and health sciences are becoming more quantitative (8), and this can revolutionize the way clinical decisions are made. In fact, effective approaches of data integration, e.g., clinical data and genomic data from patients are crucial for fast growing areas of research, such as precision and personalized medicine. This opens the opportunity of best diagnosis of diseases.

Second, if data are a primary source of scientific research, it is legitimate to recognize its value by, for example, publishing it as a peer-reviewed and citable paper. Reproducibility can increase if data are available and the methods and protocols are published in detail. The classical research paper does not include materials and methods with enough detail and data might not be fully available. Data deposition has the potential to amplify the outreach of published data and, therefore, increase the scientific reputation of the data creators (9, 10). In fact, a game theoretical analysis shows that sharing data with the community can be the most profitable and stable strategy (11). Moreover, publications with open data have higher citation rate than those with closed data (10, 12, 13).

At this point, it is important to distinguish between data deposition in public repositories and data publishing in a data journal. Data repositories are designed for data storage and retrieval. Key features include data curation, data preservation, and stewardship, as well as the promotion of the FAIR principles. Data journals, in turn, often require a structured description of the data set in terms of, e.g., motivations and used methods, as well as the deposition of the data in a specific repository. While data journals normally require data deposition, the reverse does not apply. In both situations, scientists must be aware of which license options the data journal and the repository of choice offer.

Third, society invests in science through public funded projects or charity. Data sharing is a way of returning back this investment. It reduces the duplication of experiments—thus saving resources—and allows data re-analysis from a new perspective. This is achieved either by posing new questions to the data or by repeating one same question using a different analysis. Data sharing can, in the long run, bridge the gap between labs running short on money and those that have more financial and technical resources available.

Last but not least, in health emergencies, such as the Ebola or Zika virus outbreak, society can profit from the timely access to shared data (14). Moreover, the World Health Organization advocates a paradigm shift in data sharing during such emergency outbreaks: from the embargo imposed by publication schedules, to open data in pre-publication and sharing platforms (15).

In the following sections, I present the features and guidelines that can improve data reusability.

## Features and Guidelines to Share Research Data

### SMART Experimental Design Boosts Data Reusability

SMART is the acronym for specific, measureable, attractive (or achievable), relevant, and timely (16). This approach helps to define if an idea is feasible or not, in terms of time and resources. Whether planning a wet or dry lab experiment, it is paramount to invest time and energy on the experiment design before going hands-on. Having passed this test, scientists define their experiment as simple as possible and as complex as necessary. A sound data management plan will describe the whole process of data treatment during and after a project (17). It will also account for a longer life cycle of research data, extending their value to the community (17, 18). This process includes not only the details about data generation, processing, and the quality control policy but also the data preservation and sharing plans.

This helps with the process of sharing data, because a well-designed experiment will be easier to understand and be reused by other members of the scientific community.

### Standard Formats Facilitate Data Exchange

Science has increased in complexity and interdisciplinarity throughout time. This means that scientific progress relies on a team of scientists from different fields. For this reason, it is crucial that these partners find a *lingua franca* to communicate among each other and to exchange data among them and among different platforms. In the specific field of systems biology, scientists can exchange data using standardized data tables for Systems Biology (SBTab) (19), and models using Systems Biology Markup Language (SBML) (20) or Biological Pathway Exchange (BioPax) (21) and can visualize the biological network using Systems Biology Graphical Notation (22) standards. Biomodels is a curated repository for computer models of biological processes that accepts models in SBML and CellML formats (23).

Using standard file formats to exchange data is preferable over using proprietary file formats, because the former can operate interchangeably in a wide range of platforms and software tools. However, choosing a standard format to describe data and metadata is not always straight forward. To help select the most appropriate (meta)data standard: http://biosharing.org is a manually curated platform that includes standards, databases, and data policies used in the life sciences (24). Biosharing evolved to Fairsharing, which provides the same services as Biosharing, but across all disciplines (http://fairsharing.org).

Ideally, scientists use existing standards adopted by the data repositories. However, when standards for an experiment type do not exist, generalized data serialization formats such as YAML Ain’t Markup Language (YAML) (25) or JavaScript Object Notation (26) can be considered.

### Rich Metadata Clearly Describes Research Data

Metadata provides the information that other researchers need to understand (and replicate) your data set. They describe and identify a data set and are able to locate a referenced resource (17). Data discoverability and reusability increase when the associated metadata completely describe an experiment. There are metadata standards available used by several repositories. Examples of metadata standards are the ISA-tab [cross-omics experiments (27)], MIAME [the minimal information about a micro array experiment (28)], and PDBML [describes the Protein Data Bank exchange dictionary and archival data files in XML format (29)].

There are other standards than those for (meta)data that can be considered in the process of data sharing, for example standards for data identification (see Unequivocal Identification of Data Sets Enhances Data Integration of this review).

### The FAIR Guideline Principles Ensure Data Transparency, Reproducibility, and Re-usability

FAIR is the acronym for Findable, Accessible, Interoperable, and Reusable. The FAIR principles (30) aim to be a guide to data producers and controllers. According to the previously cited article, FAIR data should be:

- Findable: easy to *find*, i.e., (meta)data have a unique and persistent identifier (PID); metadata clearly identify and richly describe the data they refer to; and (meta)data are deposited in a findable repository.
- Accessible: (meta)data are identified using standard and open protocols; metadata are accessible, even if the data no longer exist.
- Interoperable: (meta)data allow the exchange between platforms and are machine readable; (meta)data are FAIR; and refer to other sources of (meta)data, when necessary.
- Reusable: (meta)data are carefully and completely described; (meta)data have a clear and accessible license; (meta)data comply with the community driven standards.

The FAIR principles stewardship group updates a living document for the elaboration and update of these principles (available at: http://datafairport.org/fair-principles-living-document-menu).

To make a data set compliant with the FAIR principles can be a complex process. However, this is an essential step in the process of data sharing, because the FAIR principles maximize the added value of open access data and ensure transparency, reproducibility, and reusability.

### Computer Code Automatizes Monotonous Processes

Writing specific computer code automatizes monotonous process tasks and/or creates a pipeline analysis to apply to research data. This systematizes data organization and analysis. It will not only save time but will also allow keeping track of the process analysis. To make this process efficient, it is important to describe and keep track of the different versions of the computer code and the results thereof. Make certain that the computer code is efficient and reproducible by following the ten simple rules for reproducible computational research laid out by Sandve et al. (31). There are several open source programming and scripting languages, such as R (32) or Python (33), which can process and integrate data. And, more specifically, tools such as knitr (34) will allow the generation of a dynamic data-report, as well as to embed computer code into other applications, such as LaTeX. IPython (35) is an interactive computer shell that allows dynamic data visualization and provides a kernel for Jupyter. This tool is language-agnostic and supports scientific computing across many different programming languages (36). Further on, Dexy allows the documentation and maintenance of one project with one tool (37). To work platform independent, scientists can use Docker. Docker is an open source project that enables an operating system level virtualization using containers. Once the server is configured, software runs with that specific configuration—regardless of the environment—and disk images can be shared amongst cooperators (38, 39).

The automation of data analysis simplifies the process of finding, changing and identifying specific parameters of a data set and can facilitate the work with cooperators. However, automation processes can also propagate errors; therefore, human double checking is critical to assure algorithms’ robustness.

### Data Licensing Guides Future Reusers

Data licensing clearly defines how and in which conditions the data can be reused and guides future reusers (40). The Creative Commons (41) and the Open Data Commons (42) guide the subsequent use of intellectual products, such as research data. The Creative Commons offers a number predefined licenses (5) and choosing one available license is encouraged, instead of creating one. This allows defining which data sets to share, with whom, and how the data can be reused. Before attributing a license to a data set, it is important to check if the funding agency, the data repository, or the publisher applies restrictions to data licensing as a condition of funding, depositing, publishing, or as a matter of local policy. It is a good procedure to contact the institutional data management office or library to be sure which license to use.

This is especially important because there are critical situations where research data is protected by law. Clinical trials (43), patients’ data, genomic data, or results from questionnaires are sensitive data that relate to a natural person and, thus, are protected by law. In Europe, the Regulation (Eu) 2016/679 of the European Parliament and of the Council of 27 April 2016 (44) regulates the protection of personal data, which are the data that can identify a natural person directly or indirectly. However, in the case of genomic studies, data have an inherently identifying nature and, theoretically, cannot be totally anonymized, but privacy protection can avoid the misuse of the data (7). Scientists dealing with such sensitive data are advised to contact the institutional ethics office to know how to proceed. The International Committee of Medical Journal Editors proposes and supports the responsible share of de-identified individual-patient data (45).

### Unequivocal Identification of Data Sets Enhances Data Integration

Data set identifiers identify the data set and do not relate to data privacy protection. Data set identifiers should be persistent, unique, compliant with existing standards, and accepted by the specific research community. This will provide the data with a timeless identifier—even if the URL or the physical repository changes the address (46)—and will promote data integration in specific infrastructures (47). In Europe, the e-PIC consortium has been established to provide (and maintain) a PID to research data, which is unique and timeless (48). Another commonly used digital identifier is the Digital Object Identifier (DOI) (49), which not only persistently and unequivocally identifies their object but also attributes a URL to (at least) the metadata of their object. In this case, the DOI will be closely related to the metadata, and this will increase data *interoperability* between humans and/or machines.

### Data Sharing Platforms Open Up Research Data

The next step is to choose a data repository that is persistent, curated, and recognized by the scientific community. Here is a check list [extended and adapted from the checklist of the Digital Curation Centre (50)] that guides through the decision of choosing a data repository:

1. Does it require FAIR (meta)data?
2. Is it recognized by their community?
3. Can they restrict access to the data? (e.g., password protection)
4. Does it have efficient data encryption methods?
5. Does it provide good technical assistance and how much does it cost?
6. Does it curate their (meta)data?
7. Is their (meta)data citable and can they track usage and citations?
8. Can they link the data to another repository?

The re3data.org registry, to date, lists and identifies 1,500 repositories for research data (51). Datamed.org is a data search engine prototype that aims at data discovery across data repositories (52).

Normally, publishing a data set in a data journal requires sharing the respective data set in an established repository.

### Peer-Reviewed Data Journals Are a Formal Platform to Publish Scientific Data

Data journals have been identified as a key resource to promote data sharing (53). These peer-reviewed journals follow the model of standard scholarly publication and describe findable and accessible data sets by means of a metadata document (54).

There are several options to publish data. Scientists can choose preprint servers, such as arXiv (55), bioaRxiv (56), open access journals that foster the access to the data underlying the results, such as F1000 (57), or pure data journals, such as Scientific Data (58).

A survey on more than 100 currently existing data journals describes their approaches for data set description, availability, citation, quality, and open access (53).

### Integration of Data Sharing Costs in Funding Applications

The process of preparing research data to share in a sustainable way is costly in terms of time, money, and resources (59, 60). Because this investment does not have an immediate return, many researchers might be reluctant to prepare their data to share. Including data management and sharing when applying for funding is a way to overcome this limitation. This informs funders about the importance of funding sustainable data warehousing structures (60). Scientists clearly state the costs of producing FAIR data (30), of storing and publishing their data set and, very importantly, of gaining the *expertise* to perform those tasks. For that, they can hire and/or train a data scientist. There is the need to create the position of data scientist in teams dealing with research data. Therefore, the training of junior researchers and the definition of career tracks for bioinformaticians/data scientists (61) is an important asset to overcome the need of expertise in data sharing. Funders and regulators must be aware of the importance of data science in other fields of research, such as medicine, health, or biology, to name just a few. It is at the researcher’s hands to inform them of the challenges and opportunities of data sharing.

## Outlook

Scientific progress builds on the results researchers can understand. Transparency is essential to make science more understandable to others. Making research data available within and across areas increases transparency and reproducibility of scientific results. Therefore, data sharing paves the way for a more open, ethical, and sustainable science.

## Author Contributions

ASF conceived and designed the study, performed the literature research, wrote, and reviewed the manuscript.

## Conflict of Interest Statement

The author declares that the research was conducted in the absence of any commercial or financial relationships that could be construed as a potential conflict of interest.
